# Translational insights into the hormetic potential of carbon dioxide: from physiological mechanisms to innovative adjunct therapeutic potential for cancer

**DOI:** 10.3389/fphys.2024.1415037

**Published:** 2024-07-17

**Authors:** João Francisco Pollo Gaspary, Lee Edgar, Luis Felipe Dias Lopes, Carmen Brum Rosa, Julio Cezar Mairesse Siluk

**Affiliations:** ^1^ Postgraduate Program in Production Engineering, Federal University of Santa Maria, Santa Maria, Brazil; ^2^ Elastro Crete, LLC. Research and Development Department, Veyo, UT, United States; ^3^ Department of Administrative Sciences, Federal University of Santa Maria, Santa Maria, Brazil; ^4^ Production Engineering Department, Federal University of Santa Maria, Santa Maria, Brazil

**Keywords:** carbon dioxide therapy, acidic microenvironment modulation, oxygenation in cancer cells, hypercapnia in cancer treatment, pH regulation in tumor cells, immunomodulation in oncology, hormesis in cancer therapy, CO_2_-induced adaptive responses

## Abstract

**Background:**

Carbon dioxide (CO_2_), traditionally viewed as a mere byproduct of cellular respiration, plays a multifaceted role in human physiology beyond simple elimination through respiration. CO_2_ may regulate the tumor microenvironment by significantly affecting the release of oxygen (O_2_) to tissues through the Bohr effect and by modulating blood pH and vasodilation. Previous studies suggest hypercapnia (elevated CO_2_ levels) might trigger optimized cellular mechanisms with potential therapeutic benefits. The role of CO_2_ in cellular stress conditions within tumor environments and its impact on O_2_ utilization offers a new investigative area in oncology.

**Objectives:**

This study aims to explore CO2’s role in the tumor environment, particularly how its physiological properties and adaptive responses can influence therapeutic strategies.

**Methods:**

By applying a structured translational approach using the Work Breakdown Structure method, the study divided the analysis into six interconnected work packages to comprehensively analyze the interactions between carbon dioxide and the tumor microenvironment. Methods included systematic literature reviews, data analyses, data integration for identifying critical success factors and exploring extracellular environment modulation. The research used SMART criteria for assessing innovation and the applicability of results.

**Results:**

The research revealed that the human body’s adaptability to hypercapnic conditions could potentially inform innovative strategies for manipulating the tumor microenvironment. This could enhance O_2_ utilization efficiency and manage adaptive responses to cellular stress. The study proposed that carbon dioxide’s hormetic potential could induce beneficial responses in the tumor microenvironment, prompting clinical protocols for experimental validation. The research underscored the importance of pH regulation, emphasizing CO_2_ and carbonic acid’s role in modulating metabolic and signaling pathways related to cancer.

**Conclusion:**

The study underscores CO_2_ as vital to our physiology and suggests potential therapeutic uses within the tumor microenvironment. pH modulation and cellular oxygenation optimization via CO_2_ manipulation could offer innovative strategies to enhance existing cancer therapies. These findings encourage further exploration of CO2’s therapeutic potential. Future research should focus on experimental validation and exploration of clinical applications, emphasizing the need for interdisciplinary and collaborative approaches to tackle current challenges in cancer treatment.

## 1 Introduction

Carbon dioxide (CO_2_), a basic metabolic byproduct of cellular respiration, has important roles beyond simple elimination through the lungs. Despite its small quantity, it greatly influences the efficiency of the respiratory system and aerobic metabolism. Through the Bohr effect, CO_2_ affects oxygen’s (O_2_) release to tissues, thereby altering hemoglobin’s affinity for O_2_. This, along with its role in adjusting blood pH and cardiovascular functions such as vasodilation and blood pressure, highlights its physiological significance (Hall and Hall, 2020; Feinstein et al., 2022; Doyle and Cooper, 2024). In the past, CO_2_ has been linked to various therapeutic techniques, including as one of the first anesthetics (Bohr et al., 1904; Rose, 1905; Henderson, 1940; Buess, 1953). Currently, statements such as Henderson’s (1940) claim, “CO_2_ is the primary parahormone of the whole body; it is the only one produced by all tissues and likely impacts every organ. CO_2_ is, in fact, a more essential component of living matter than O_2_,” are being reexamined in modern medicine. This underscores a renewed interest in understanding the various aspects of CO_2_, from its essential physiological functions to its potential negative implications. This has led to ongoing debates considering whether CO_2_ is a friend or foe in the context of human health.

Beyond its physiological role, CO_2_ has been studied in extreme situations where human adaptation to high CO_2_ levels, such as those experienced by deep-sea divers and mountaineers, showcases the body’s capacity to adapt to hypercapnia conditions. Studies show that this adaptation can lead to improved efficiency in O_2_ usage, indicating optimized cellular mechanisms for O_2_ delivery and use under stress (Florio et al., 1979; Earing et al., 2014; Bailey et al., 2017). This shows the human respiratory and cardiovascular system’s flexibility and resilience under intense physiological challenges.

These findings parallel the tumor microenvironment, characterized by hypoxemia, alkalosis, acidosis, and intense cellular stress (Bożyk et al., 2022). Human adaptability to high CO_2_ levels suggests possible strategies to manipulate the tumor microenvironment to enhance current treatments and devise new ones. Given the tumor microenvironment’s complexity and the central role of cellular stress in tumor growth (Redding and Gabocka, 2023), investigating the hormetic potential of CO_2_—the ability of stress at optimal levels to induce a beneficial response—becomes a promising area of investigation. This concept underscores the need to understand the fundamental biology behind the human body’s responses to CO_2_. This is especially important given that most therapies targeting the tumor microenvironment, excluding immunotherapies, continue to face challenges in proving efficacy (Xiao and Yu, 2021).

In line with this understanding, this study aims to analyze the role of CO_2_ in the context of the tumor microenvironment. Specifically, it will focus on how the physiological properties of CO_2_ and adaptive responses can be applied or simulated in therapeutic strategies. It seeks to uncover new approaches through an enhanced understanding of CO_2_’s complex role in cellular physiology and its impact on tumor cell behavior. This comprehensive backdrop guides our methodological strategy, designed to dissect CO_2_'s nuanced roles within the tumor microenvironment and uncover groundbreaking therapeutic avenues.

## 2 Methods

This detailed understanding of CO_2_’s physiological roles sets the stage for our methodology, where we employ a structured translational approach to delve deeper into CO_2_'s interaction with the tumor microenvironment, aiming to uncover its therapeutic potential. Translational Research was employed, using the Work Breakdown Structure (WBS) method as advised by the Project Management Institute. The translational research method, detailed below, is meticulously devised to unravel the intricate interactions of CO_2_ within the tumor microenvironment, directly addressing the inquiries posed in the Introduction and laying a foundation for the ensuing analysis. This approach subdivided the research process into six cohesive Work Packages (WPs), each addressing specific facets of CO_2_ interaction with the tumor microenvironment for a comprehensive, interdisciplinary view. A detailed qualitative assessment of the results will follow each WP to confirm their clinical relevance and application.

The goal of WP1 (Management and Supervision) was to run the project efficiently, adhere to the predefined goals and timeline, stimulate collaboration by utilizing Open Innovation to review and adjust each WP as the project unfolds, ensure effective communication among the WPs, and evaluate the significance of new medical hypotheses.

**FIGURE 1:**
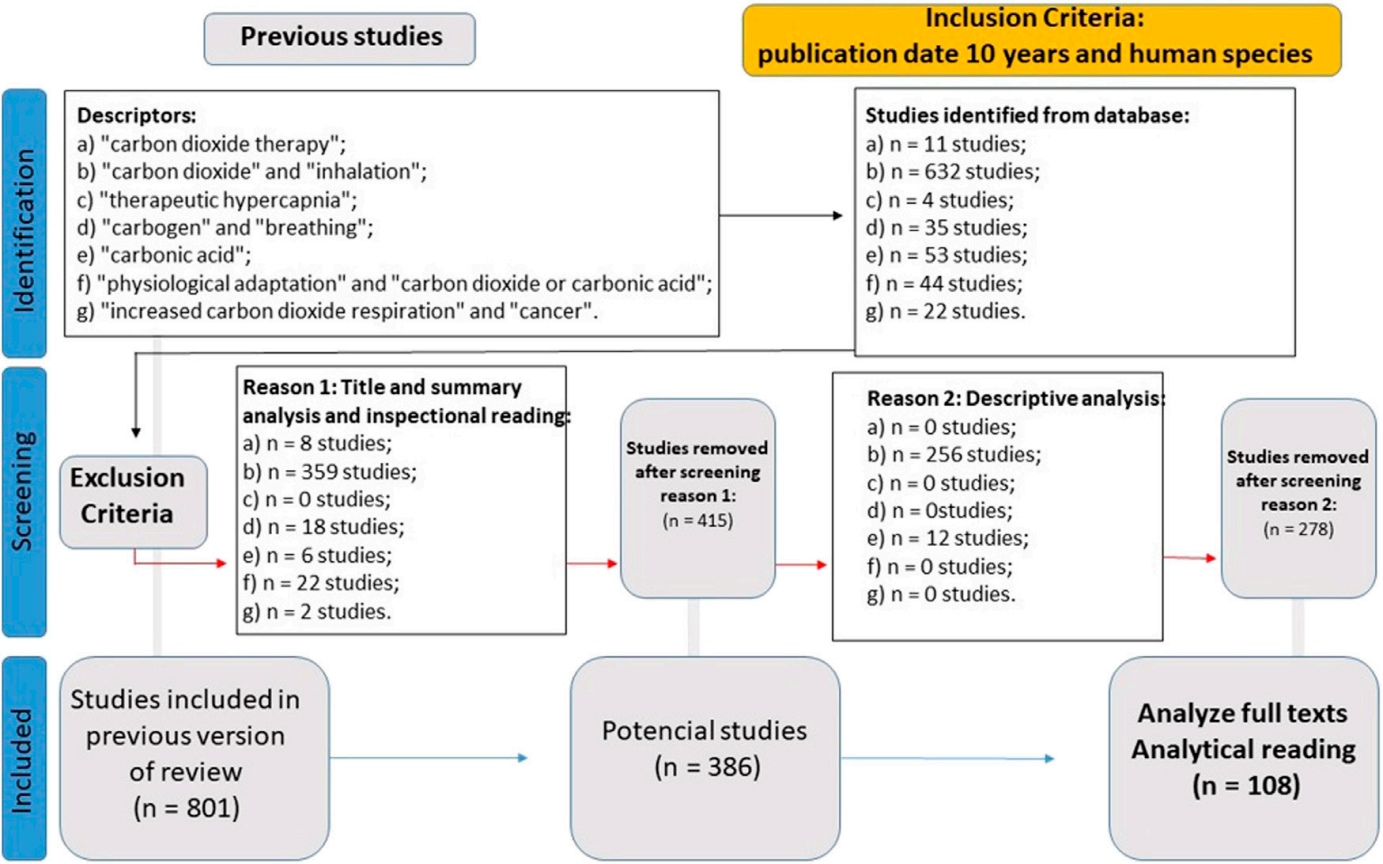
Flowchart of the systematic literature review in WP2.

WP2 (CO_2_ Data Analysis) aimed to produce a current theoretical framework on CO_2_. This aim was achieved through a systematic literature review, which gathered data from the past decade in relation to the human species with descriptors such as “carbon dioxide therapy,” “carbon dioxide and inhalation,” “therapeutic hypercapnia,” “carbogen and breathing,” “carbonic acid,” “physiological adaptation and carbon dioxide or carbonic acid,” and “increased carbon dioxide respiration and cancer.” The results amounted to 108 articles for thorough analysis (Figure 1).

**FIGURE 2:**
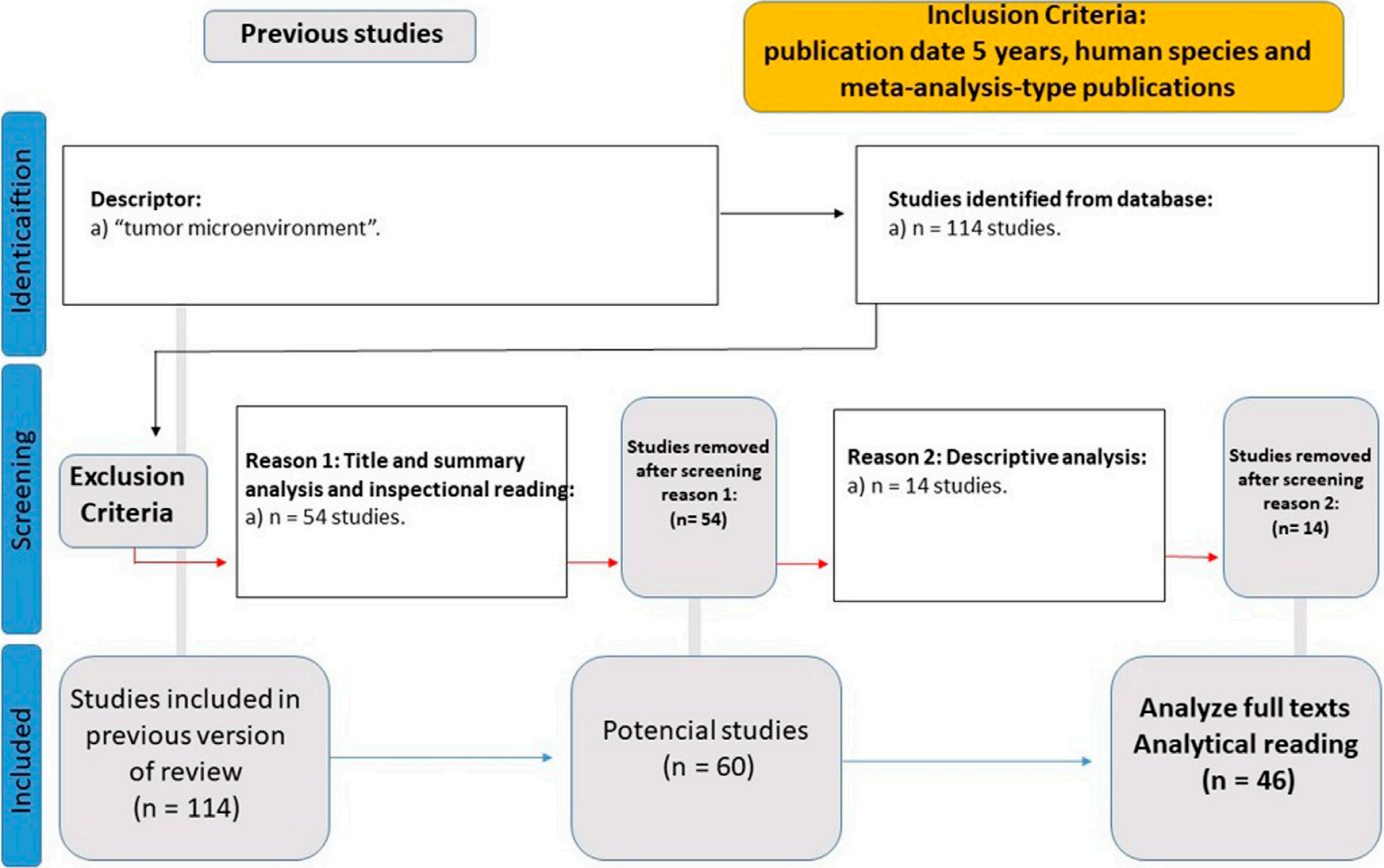
Flowchart of the systematic literature review in WP3.

The specific objective of WP3 (Tumor Microenvironment Data Analysis) was to select primary study targets influencing new cancer therapies and prepare for WP4. This led to the selection of Fundamental Viewpoint Factors according to the multi-criteria decision approach of Bana e Costa et al. (1999). Another systematic literature review was conducted with descriptors, such as “tumor microenvironment,” using filters for the past 5 years, human species, and meta-analysis-type publications. The output was 46 articles for detailed analysis (Figure 2).

The primary aim of WP4 (Data Integration) was to identify Critical Success Factors (CSFs) for merging knowledge obtained from prior WPs based on a multicriteria decision-support method inspired by Bana e Costa et al. (1999). This process was vital for data synthesis and interpretation, establishing a foundation for practical applications. The CSFs were then authenticated through an integrative literature review (Table 1), with selection criteria adapted from Khan et al. (2011), which emphasized the wide-ranging inclusion of translational data.

**TABLE 1:** Article selection criteria utilized for this study in WP4.

Criteria	Description
Database	LILACS; Medline; Web of Science; Scopus; SciELO; Google Research; Research Gate
Time Limit	2014 2024
Languages	English, Portuguese, or Spanish
Indexed Terms	English descriptors generated from CSFs derived from free association
Inclusion Criteria for Analysis	Broad, in order to include data for translational research

Based on data from Khan et al. (2011).

The key objective of WP5 (Modulation of the Extracellular Environment) was to investigate how integrating theory and data could impact the tumor’s microenvironment. Design Thinking and Open Innovation were used to bridge theoretical discoveries with practical applications (Chesbrough, 2003; Brown, 2008). This phase includes a plan for empirically validating the findings through case studies.

The primary goal of WP6 (Publication of the Results) is to assess the outcomes’ inventiveness and applicability using SMART criteria (Drucker, 1954) and FINER criteria (Hulley et al., 2007) to create innovative clinical solutions. Additionally, we will address the methodological restrictions and ethical elements related to the study, especially concerning the use of human patient data, to ensure the study’s transparency and repeatability. This methodological design signifies an adherence to accuracy, repeatability, and ethics in research that lays a strong groundwork for investigating the therapeutic possibilities of CO_2_ in cancer treatment. Our structured analytical journey, outlined in the methodology, paves the way for the findings we present. These results not only validate our investigative approach but also open new vistas in understanding CO_2_'s therapeutic dimension.

## 3 Results

Applying the structured approach outlined, we systematically explored CO_2_'s multifaceted impact on the tumor microenvironment. The following results illuminate the adaptability of human physiology to CO_2_ variations and its implications for therapeutic strategies. The findings presented herein vividly illustrate the influence of CO_2_ and carbonic acid on cellular physiology and the tumor microenvironment, directly correlating with our methodological objectives and bridging the gap to the comprehensive discussion that follows. To optimize the presentation of the results, the key points obtained through WPs one to five are listed in Table 2.

**TABLE 2:** Key methodological points.

WP	Actions	Keypoints
1	Management and Supervision	The goal was to transiently optimize the body’s pH in pursuit of equilibrium in bodily homeostasis and to increase the human body’s zeta potential
2	CO₂ Data Analysis	Completed. This will present the potential contributions to the tumor microenvironment
3	Tumor Microenvironment Data Analysis	The complexity of the tumor microenvironment and the metabolism of cancer cells were identified; Understanding the need for a more integrated and multifaceted approach to comprehend and treat cancer; Selection of fundamental viewpoints
4	Data Integration	Identification and validation of critical success factors
5	Modulation of the Extracellular Environment	Formulation of a medical hypothesis on how the hormetic potential of CO₂ could contribute to localized action in the tumor microenvironment; Suggestion of a clinical protocol

For WP2, it is crucial to understand the basic technical knowledge needed to grasp the potential of biological and therapeutic interaction with carbon dioxide, covered across various topics in Table 3.

**TABLE 3:** List of basic technical knowledge required for understanding the biological and therapeutic interaction of CO₂.

Topic	Description	Authors
Vasodilation	CO₂ can induce vasodilation or the enlargement of blood vessels. This enhances tissue oxygenation and nutrient delivery and facilitates the removal of metabolic waste	Moris et al. (2023); Sur et al. (2024)
Bohr effect	CO2 presence in tissues boosts O2 release from hemoglobin to tissues, by reducing hemoglobin’s O2 affinity, thereby enhancing oxygenation in tissues requiring more O2	Benner et al. (2024)
Neoangiogenesis Stimulation	Exposure to CO_2_ can promote neoangiogenesis, the formation of new blood vessels. This is especially advantageous for wound healing and aesthetic procedures because it improves vascularization and supports skin regeneration	Moris et al. (2023)
Collagen Production Stimulation	CO₂ therapy is shown to boost collagen production, leading to better skin elasticity and appearance by improving blood flow and oxygenation, which stimulates collagen synthesis in skin cells	Bagherani et al. (2023); Takano et al. (2023)
Transcutaneous Therapies/Carboxytherapy	CO₂ is widely used in therapeutic applications within aesthetics and medicine, treating skin conditions such as cellulite, stretch marks, and scars, and improving vascular health	Bagherani et al. (2023)
Therapeutic Hypercapnia	Hypercapnia may stabilize brain and lung tissue, potentially via pH modulation. This suggests hypercapnia can influence cellular function by altering the extracellular environment	Stetter et al. (2021); Marongiu et al. (2021)

Furthermore, WP2 underscores the use of carbogen (a gas mixture comprising approximately 5% CO_2_ and 95% O_2_) to augment cancer treatments. This blend is employed in numerous medical and scientific scenarios due to its unique properties that are advantageous for diagnosing and treating many conditions, as outlined in Table 4.

**TABLE 4:** Some applications and benefits of carbogen.

Indication	Description	Authors
Support in Radiotherapy	Incorporating CO₂ into the gas mixture may enhance blood oxygenation, potentially increasing the sensitivity of cancer cells to radiotherapy. It is suggested that this rise in tissue oxygenation boosts the effectiveness of radiation therapy for specific cancer types	Leavitt et al. (2024)
Treatment of Vascular Disorders	Carbogen is used to treat certain blood circulation conditions, including vascular disorders of the retina. Enhanced oxygenation promotes vascular health and minimizes tissue damage	Shariffi et al. (2023)
Respiratory Function	The carbogen mixture is utilized in physiological studies to evaluate the respiratory system’s reaction to CO₂ level fluctuations. This assists in the diagnosis and monitoring of conditions that impact breathing	Krasniqi et al. (2020); Holick (2022)
Brain Imaging Studies	In neuroscience, carbogen is utilized to enhance cerebral oxygenation and improve the quality of images obtained	Naik et al. (2023)

Regarding WP3, it is noteworthy to cite studies examining the tumor microenvironment, particularly those focusing on tumor metabolism (Benedetti et al., 2023; Luo and Liang, 2023; Micalet et al., 2023). These studies pay attention to the regulation of pH levels in tumor cells, including the distinct expression of ion transporters and proton pumps that may be more active or differently regulated in cancer cells compared to healthy ones (Qiao et al., 2017; Khalaf et al., 2021). Additional research shows that cancer cells’ intracellular pH (pHi) differ from normal cells. Typically, normal cells have a lower pHi than the extracellular pH (pHe), with pHi and pHe values ranging mainly from 7.0 to 7.2 and 7.3 to 7.4, respectively. Conversely, cancer cells tend to have an elevated pHi, between 7.12 and 7.65, and a reduced pHe range of 6.2–6.9, a reversal in intra-extracellular pH gradient indicative of cancerous tissue that promotes disease progression (Persi et al., 2018). This phenomenon, represented by extracellular acidosis and intracellular alkalinization, helps trigger cellular proliferation, evade apoptosis, initiate a metabolic shift to aerobic glycolysis (the Warburg effect), foster genetic instability, and potentially enhance multi-drug resistance within cancer cells. Increased pHi and decreased pHe can also augment invasion and metastasis. Hence, assessing the pHi in tumors could offer a promising way to monitor cancer progression and responses to treatment (Shirmanova et al., 2015). However, due to methodological limitations, there could be subject to vast variations (Table 5).

**TABLE 5:** Limitations in measuring intracellular pH.

Topic	Description
Limitations of the Current Techniques	Methods for measuring intracellular pH (pHi), including fluorescence pH probes, nuclear magnetic resonance, and pH microelectrodes, face challenges in accuracy, resolution, and potential cellular disruption. These limitations may hinder the ability to fully capture pHi variations, especially in complex *in vivo* conditions, potentially underestimating the pH discrepancy between tumor and normal cells
Intracellular and Inter-cell Variability	Within a single cell, pH levels can significantly differ across compartments like mitochondria, cytosol, and nucleus, and there’s notable variation among cells in the same tissue or culture, which current measurement methods may not fully capture
Effects of the Microenvironment	The cellular microenvironment, particularly in tumor tissues, can affect pHi in challenging ways to reproduce *in vitro*. Interactions between cells, the extracellular matrix, and soluble factors can significantly influence pHi, which standard culture conditions might not accurately replicate

Based on data from Loiselle and Casey (2010) and Jaworska and Malek (2021).

For WP3, it is also worth highlighting the Fundamental Viewpoint Points (FVPs) that were chosen based on predefined parameters related to the tumor microenvironment, as seen in Table 6.

**TABLE 6:** Key viewpoints selected in physiology and cancer biology support possible discrepancies in intracellular pH between healthy and oncological cells.

FPV	Characteristics
Altered Energy Metabolism in Tumor Cells (Bader et al., 2020; Dey et al., 2021; Yang et al., 2023)	Cancer cells frequently show the Warburg effect, preferring anaerobic glycolysis for energy, even with available O₂, leading to lactic acid production and a possibly acidic extracellular environment. Despite this, they keep an alkaline intracellular pH, suggesting they have more robust pH regulation mechanisms than healthy cells
Adaptations for Survival and Proliferation (Boedtkjer and Pedersen, 2020; Wang et al., 2020; Li et al., 2021)	Maintaining an alkaline intracellular environment can promote cancer cell survival and proliferation under stressful conditions. This enhances enzyme activity for metabolism and DNA synthesis and offers resistance to certain cell death types
Tumor Heterogeneity (Kudelova et al., 2022; Zhang et al., 2022; Chhabra and Weeraratna, 2023).	Tumors display remarkable heterogeneity in cell types and microenvironments, affecting O₂ and nutrient availability. This leads to varied cellular metabolism and pH regulation across the tumor, indicating that pHi in tumor cells may fluctuate more than in uniform healthy tissues

With respect to WP4, Yew Wong and Aspinwall (2005) classified the CSFs due to their comparable approach to the FVPs. The CSFs, presented in Table 7, represent areas of significant integration between the tumor microenvironment and the biological impact of CO_2_/carbonic acid, which is derived from the integration process of the previously defined FVPs.

**TABLE 7:** CSFs.

CSF	Description	Action potential of the biological influence of CO₂/Carbonic acid
Inwardly Rectifying Potassium Channels (Kir) (Putnam et al., 2004; Zhang et al. (2020a), Xia et al. (2023))	Kir channels stabilize resting membrane potential and cell volume by favoring K⁺ influx, aligning the membrane potential with potassium’s equilibrium	Changes in CO₂/carbonic acid levels modulate Kir channel activity, impacting action potentials and cellular functions like muscle and neuronal activities
Calcium/calmodulin-dependent protein kinases (Huang et al., 2021; Zhan et al., 2022)	An enzyme essential for intracellular signaling is activated by binding to calmodulin, a protein that attaches to calcium (Zhang et al., 2022)	Alterations in CO₂/carbonic acid levels influence calmodulin kinase signaling in the CNS and other pH-sensitive tissues, impacting biological action potentials
Carbonic Anhydrase IX (Angeli et al., 2020)	The emphasis is on how hypoxia aids tumor growth and the importance of amino acid and pH balance, underlining carbonic anhydrase’s role in managing O₂ and CO₂ levels in the tumor environment	Carbonic anhydrase activity in tumors regulates pH by converting CO₂, affecting cancer cell metabolism and pathophysiology beyond simple gas balance, indicating a significant influence on action potentials. (Venkateswaran and Dedhar, 2020)
Extracellular Acidic Environment (Boedtkjer and Pedersen, 2020)	The tumor microenvironment is complex due to increased lactic acid production and cancer cells’ active removal of protons, leading to extracellular acidification	Introducing carbonic acid into an acidic environment triggers a neutralization reaction, turning CO₂ into bicarbonate and protons, thus buffering extracellular acidity. This modification influences tumor progression and treatment response by affecting the action potential through altered pH dynamics
Mitochondrial Dysfunction (Wang et al., 2022)	This dysfunction is involved in numerous diseases, including cancer. It impacts cellular metabolism, potentially leading to a pro-inflammatory state and tissue damage. (Menchikov et al., 2023).	Mitochondrial dysfunction alters cancer metabolism (Warburg effect), and extracellular pH changes, driven by CO₂/carbonic acid levels, impact tumor cell invasion, migration, and therapy resistance, affecting biological action potentials
H⁺ ATPases (Liu et al., 2021; Qi et al., 2022), and Na⁺/H⁺ Exchangers Zhou et al. (2021), Liao et al. (2024)	H⁺ ATPases and Na⁺/H⁺ exchangers have critical roles in the tumor microenvironment. They contribute to unique cancer characteristics, including invasion, metastasis, and therapy resistance	The coordinated activity of H⁺ ATPases and Na⁺/H⁺ exchangers, influenced by CO₂/carbonic acid levels, drives a cycle of extracellular acidification and intracellular pH stabilization, aiding tumor survival, invasion, metastasis, and therapy resistance. Understanding these dynamics is crucial for developing therapies targeting the tumor microenvironment’s pH to combat cancer
Cell Membrane Instability (Fan et al., 2021; Zhu et al., 2023)	Several factors impact the cell membrane’s stability. These include lipid composition, cholesterol presence, membrane proteins, and the cytoskeleton	Changes in CO₂/carbonic acid levels affect membrane fluidity, barrier selectivity, and molecule transport, impacting the biological action potential and cellular functions
Extracellular Vesicles (EV) Parayath et al. (2020), Gondaliya et al. (2023)	EVs, encompassing exosomes and microvesicles, are deliberately released by cells, including cancer cells, for extracellular communication, carrying proteins, lipids, and nucleic acids from healthy and malignant origins	CO₂/carbonic acid levels influence cancer cells’ use of EVs to modify their environment, promoting angiogenesis, immune evasion, and metastasis. EVs facilitate genetic and protein exchanges that boost tumor survival and spread, including mechanisms for drug resistance, thus affecting the biological action potential and cancer therapy outcomes
Ionic or Gas Leaks (Bożyk et al., 2022)	Ion and gas leakage into the extracellular space affects pH, signaling, membrane potential, and immune reactions within the tumor environment, impacting cancer progression, angiogenesis, invasion, and metastasis	Balancing ion transporter activity, influenced by CO₂/carbonic acid levels, is essential to prevent ionic leakages that threaten cell integrity. This regulation ensures proper osmolarity and volume, affecting the cell’s action potential and stability
Reduction in CO₂ Production by Cancer Cells due to the Warburg effect (Bożyk et al., 2022)	The Warburg effect, a metabolic shift in cancer cells to aerobic glycolysis, produces lactate instead of CO₂, allowing adaptation to hypoxia and affecting CO₂ output	Cancer cells’ preference for aerobic glycolysis, leading to variable CO₂ production across tumors, reflects the impact of CO₂/carbonic acid levels on the tumor microenvironment. This metabolic shift to lactate production affects biological action potentials and cellular behavior differently than mitochondrial respiration
Cellular Zeta Pontential (Mendivil-Alvarado et al., 2023; Hughes, 2024)	The zeta potential measures the electrical potential of particle surfaces in suspension, indicating the colloidal stability of these particles in a solution	The acidity influenced by CO₂/carbonic acid levels can modify the surface charge and zeta potential of EVs and target cells, altering their interactions, binding, and internalization capabilities. This regulation of EV effectiveness in transmitting biological information like microRNAs or proteins affects cell signaling and action potentials within the tumor microenvironment
Heme Oxygenase (Surh et al., 2020; Luu Hoang et al., 2021)	Heme catabolism, facilitated by heme oxygenase (HO), with HO-1 expression induced by stress like hypoxia, plays a role in cancer progression, impacting cell growth, apoptosis resistance, angiogenesis, and metastasis	HO-1 enzyme activity, producing CO from heme catabolism, impacts biological pathways and action potentials through signaling effects. While CO has anti-inflammatory and cytoprotective roles at normal levels, in cancer, it can enhance a tumor microenvironment conducive to cancer cell growth and survival, influenced by CO₂/carbonic acid dynamics
Autophagy (Chung et al., 2020; Kang et al., 2020; Zhan et al., 2022)	The autophagy process is intricate, involving numerous sequential steps that could intracellular pH impact	The influence of CO₂/carbonic acid on pH is pivotal for autophagy regulation, crucial for cellular health and implicated in diseases like cancer and neurodegeneration, thereby impacting biological action potentials
Gasotransmitters (Salihi et al., 2022).	The relationship between carbonic acid, nitric oxide (NO), hydrogen sulfide, CO, and the tumor microenvironment	Adjustments in CO₂/carbonic acid levels can influence blood flow and oxygenation, affecting NO generation and H₂S enzyme activity, including, including cystathionine gamma-lyase (CSE), cystathionine beta-synthase (CBS), and 3-mercaptosulfur transferase (3-MST), thereby altering their availability and effects, impacting biological action potentials and physiological responses
Intracellular Reactive Oxygen Species (ROS) (Scharping et al., 2021; Li et al., 2022)	The intracellular ROS concentration significantly influences multiple cellular processes. ROS are highly reactive molecules capable of causing oxidative harm to proteins, lipids, and DNA. However, they also serve as secondary messengers in cellular signaling pathways	CO₂/carbonic acid levels affecting intracellular and extracellular acid-base balance can alter ROS production. Acidic conditions boost ROS-generating enzyme activities like NADPH oxidase and can reduce antioxidant defenses, raising ROS levels. Mitochondrial function and the electron transport chain are impacted by pH shifts, with increased acidity heightening oxidative stress through enhanced mitochondrial ROS, influencing biological action potentials and cellular health
Chronobiological Variations (Zhou et al., 2022)	The circadian rhythm is an evolutionarily preserved timing system that governs various processes such as sleep-wake, feeding-fasting, and activity-rest cycles. It coordinates all organs’ behavior and physiological functions to maintain the body’s overall homeostasis	The human body’s pH buffering system, influenced by CO₂/carbonic acid levels, maintains a tight acid-base balance, despite daily and circadian rhythm-induced variations that affect physiological behaviors. These pH fluctuations, driven by sleep, diet, and activity patterns, can impact cancer progression, suggesting that targeting circadian and behavioral influences on pH could offer new approaches to altering the tumor microenvironment and affecting biological action potentials
Sirtuins (Katsyuba et al., 2020; Wu et al., 2022; Xu et al., 2022) and Ampk (Keerthana et al., 2023)	Sirtuins and AMP-activated protein kinase (AMPK) are critical metabolic regulators. They react to changes in the cellular environment, including nutrient availability, energy levels, and cellular stress. These enzymes are essential in preserving energy stability, promoting longevity, and resisting stress	CO₂/carbonic acid-induced pH changes can affect energy metabolism and NAD + levels, thereby influencing sirtuin activity and cellular stress responses like hypoxia, leading to alterations in acid-base metabolism. These pH shifts can activate sirtuin-involved signaling pathways and indirectly modulate AMPK activation by impacting the cell’s energy state, highlighting the role of pH in cellular adaptive mechanisms and biological action potentials
Matrix Metalloproteinases (MMPs) (Gonzalez-Avila et al., 2020; Niland et al., 2021)	Matrix metalloproteinases are proteolytic enzymes integral to the degradation of extracellular matrix components such as collagen, gelatins, elastin, fibronectin, and laminin	CO₂/carbonic acid levels influencing the tumor microenvironment’s pH can regulate MMP expression, crucial for tumor invasion and metastasis through the degradation of extracellular matrices. Understanding this pH-MMP relationship is key to developing therapies that either modify the microenvironment’s acidity or inhibit MMP activity directly, aiming to halt cancer spread by affecting biological action potentials and cellular interactions

In WP5, we hypothesized how carbonic acid could facilitate intracellular O_2_ penetration by exploring fundamental physiology and cellular biochemistry concepts. This hypothesis derives from a complex system of adaptation in which hypercapnia, via increased circulating carbonic acid, could alter cellular function and O_2_ dynamics in diverse ways. These mechanisms may explain how extracellular environment manipulation could enhance O_2_ penetration into cells, providing a fresh perspective for therapies against cancer and other pathological conditions (Table 8). Further scrutinizing these mechanisms may reveal new research pathways and therapeutic targets.

**TABLE 8:** Potential areas (Keypoints) of influence of CO₂/carbonic acid.

Keypoint	Description
Effect of pH on Hemoglobin Oxygenation State (Sekyonda et al., 2024)	The Bohr effect describes how increased CO₂ and carbonic acid levels lower blood pH, triggering more O₂ release from hemoglobin, thus enhancing tissue oxygenation in high carbonic acid environments
Cell Membrane Permeability (Alishahi and Kamali, 2019; Zhang et al., 2020b)	Gas concentration gradients and solubility influence membrane permeability to gases like CO₂ and O₂. CO₂ reacts with water to form carbonic acid, which dissociates into bicarbonate and protons, altering extracellular pH. This pH change can affect membrane protein structure and permeability, including to O₂. Additionally, aquaporin channels, which mainly transport water, might affect the movement of gases under certain pH conditions, thus influencing O₂ diffusion across the membrane
Regulation of Cellular Metabolism (Quade et al., 2021 Palmer and Clegg (2023)	Intracellular pH regulates cellular metabolism. Variations in acidic conditions can impact several metabolic pathways, including energy production and O₂ use. For instance, a mildly acidic environment could enhance the activity of some enzymes or change cellular O₂ demand
Effect on O₂ Transport (Vasodilation) Hoiland et al. (2019)	The Bohr effect enables hypercapnia to aid in releasing O2 from hemoglobin, enhancing tissue oxygenation. Hypercapnia can induce vasodilation, which improves blood flow and, in turn, enhances O2 delivery to tissues, including tumors
Metabolic Adaptation (Cummins et al., 2014; 2020)	Changes in the extracellular environment can induce cells to adapt their metabolism. This could include mechanisms that promote efficient O₂ utilization
Cellular Zeta Potential (Hughes, 2024)	The zeta potential reflects the electrical potential difference at the cell membrane, affecting colloid stability and cell interactions. Hypercapnia and resultant carbonic acid changes can modify the zeta potential, altering cellular interactions with O₂. Shifts in electrical charge distribution might create electromagnetic fields influencing polar molecules like O₂, potentially enhancing their cellular penetration
Mitochondrial Activity (Phelan et al., 2023 Quintana-Cabrera and Scorrano (2023)	Mitochondria, crucial for energy production and O₂ use, can be affected by external changes like hypercapnia, altering intracellular pH. This may impact ATP production and modify cellular O₂ demand and utilization
Non-Traditional Models of Reception and Signaling (Sarkar and Chattopadhyay, 2021).	The idea that traditional receptor models might not capture all cellular complexities suggests biophysical mechanisms, such as alterations in intracellular water structure and ion dynamics, play essential roles in O₂ penetration and use. The hydration and organization of intracellular water can influence O₂ solubility and transport, and the function of enzymes and mitochondrial proteins crucial for O₂ metabolism
Stabilization of Ionic and Gas Leaks from the Cell (Nicholls, 2021; Stahl-Meyer et al., 2021; Bertholet and Kirichok, 2022).	Membrane stability is crucial for cellular homeostasis and preventing ionic and gas leaks. Changes in the extracellular environment, like those induced by acids, can affect this stability. Carbonic acid, despite being inert, might affect lipid and protein structures in the membrane, possibly enhancing stability against leaks, especially from mitochondria. This mechanism could help maintain cell integrity under stress, including in tumors
Modulation of Inflammation and Oxidative Stress (Rivers and Meininger, 2023)	Oxidative stress and inflammation are key in progressing diseases like cancer. Managing these with molecules such as carbonic acid, by stabilizing the cell membrane and reducing nonspecific permeability, could limit the influx of ions and pro-inflammatory molecules, potentially offering a novel treatment strategy to alleviate oxidative stress and inflammation

**FIGURE 3:**
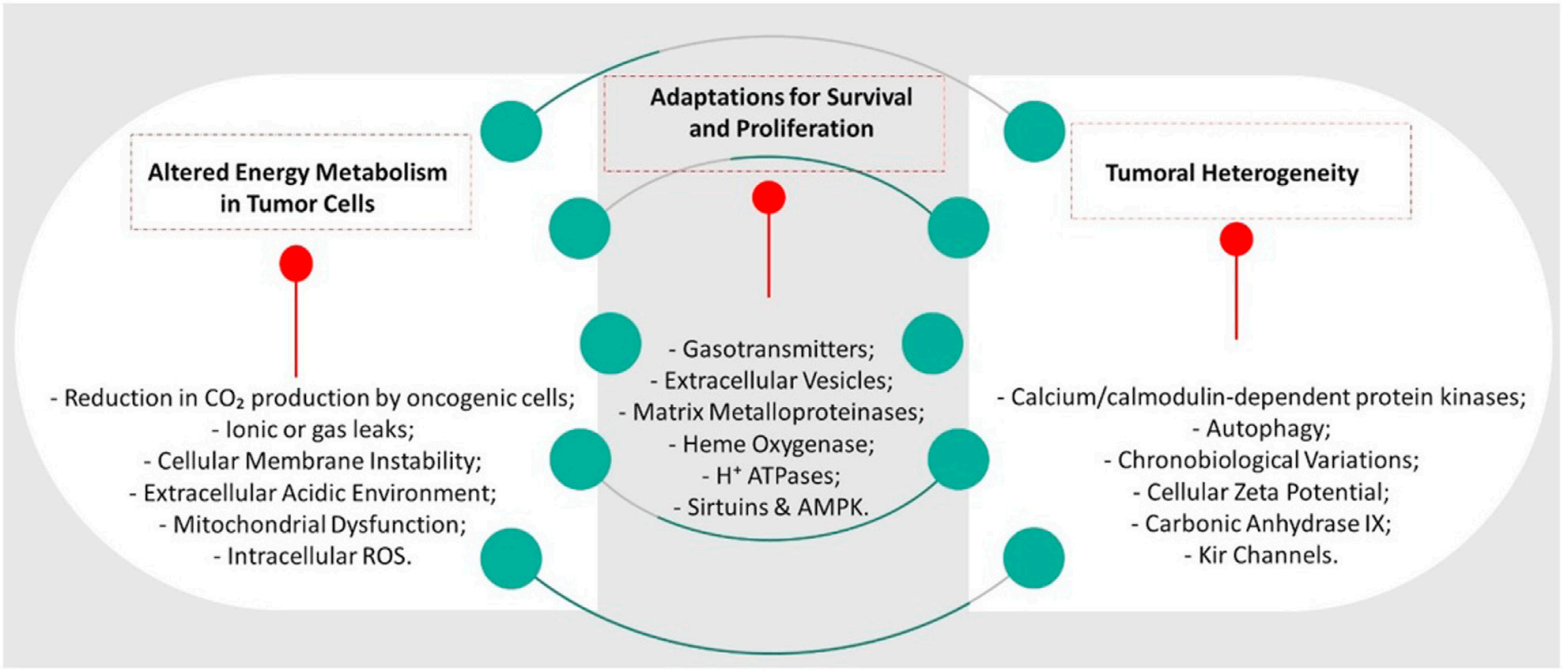
Interrelationship between FPVs and CSFs.

The tumor microenvironment typically exhibits hypoxia and acidosis, attributes that foster cancer advancement, resistance to treatment, and immune suppression (Bożyk et al., 2022). Partial restoration of homeostasis can be achieved by manipulating the microenvironment’s pH through increased concentration of carbonic acid. This impacts tumor dynamics, improves tumor cell oxygenation, alters extracellular vesicle functionality, and enhances patient survival and quality of life. Improving oxygenation in tumor tissue indirectly could heighten cancer cell sensitivity to conventional therapies such as radiotherapy and chemotherapy, a phenomenon similar to carbogen use (as per Table 4). Therefore, anticipated outcomes from clinical exploration of the hormetic effects of CO_2_ on cancer include increased tumor oxygenation, increased tumor cell sensitivity to treatments, modulation of the tumor microenvironment, and development of combined treatment strategies. Guided by these insights, this relationship is depicted through the Fundamental Viewpoint Factor and their corresponding CSFs in Figure 3.

After the study, a SMART and FINER analysis was carried out in WP6 to assess the potential impact of this novel medical hypothesis. It was scrutinized against the goals of being specific, measurable, achievable, relevant to cancer research and having a defined timeline for accomplishment (Table 9).

**TABLE 9:** Outcome of SMART and FINER application on the medical hypothesis.

Criteria	Analysis Description
Specificity (S)	The medical hypothesis clearly outlines the aims: to examine the impact of hypercapnia on tumor oxygenation, sensitize tumor cells, alter the tumor microenvironment, and devise combined treatment approaches
Measurability (M)	We can gauge these objectives through precise indicators, including the enhancement of tumor tissue oxygenation determined by imaging techniques. Other indicators consist of evaluating the sensitivity of tumor cells to therapies in laboratory and clinical models and analyzing effects on the tumor microenvironment using biomarkers for inflammation and angiogenesis
Achievability (A)	The goals seem attainable, using advancements in translational research technologies and detailed investigations in animal models and clinical trials. However, the tumor microenvironment’s complexity and cancer type differences may present substantial challenges. The necessity for interdisciplinary work and collaboration across various research fields could be a constraining factor in planning
Relevance (R)	The medical hypothesis is undeniably significant, focusing on a crucial area of cancer biology—enhancing the tumor microenvironment to amplify treatment responses. This method could potentially address recurring issues in cancer therapy, including resistance to traditional treatments and tumor progression
Timeliness (T)	While the initial plan lacked a specific timeline, applying the SMART methodology requires establishing explicit deadlines for each phase of the project. The research’s complexity and innovative nature suggest it will span several years, from basic research through to clinical trials and, pending success, to clinical application
Feasible (F)	The hypothesis is feasible using advanced translational research technologies, including *in vitro* studies, animal models, and clinical trials. However, the complexity of the tumor microenvironment and variations among different cancer types must be considered. Available resources and technologies allow for the necessary studies to test the hypothesis
Interesting (I)	The hypothesis is interesting because it explores a new therapeutic potential of CO2 in cancer treatment, a field that has not been extensively investigated. The research can provide innovative insights and open new avenues for cancer treatment
Novel (N)	The hypothesis is novel as it proposes the hormetic use of CO2 to modify the tumor microenvironment, an unconventional approach. The originality of the hypothesis can lead to discoveries that challenge current knowledge and offer new therapeutic options
Ethical (E)	The research is ethical as long as it follows established protocols for clinical and experimental studies, including informed consent from participants and appropriate ethical review. The study can be conducted ethically, adhering to international standards of medical research
Relevant (R)	The hypothesis is relevant as it addresses a significant issue in cancer therapy—treatment resistance and tumor progression. The results could have a substantial impact on clinical practice, improving outcomes for cancer patients

Our findins support the understanding that there are connections between tumor microenvironment and carbon dioxide. The connection lies in the tumor microenvironment, which is characterized by a limited extracellular buffering capacity to handle lactic acid. The connection between this and carbon dioxide (CO_2_) involves several key points (Table 10).

**TABLE 10:** Key points connection between this and carbon dioxide (CO₂).

Key point	Description
Glycolytic Metabolism and Carbon Dioxide Production	The predominant glycolytic metabolism in tumor cells significantly reduces mitochondrial activity, leading to a decrease in CO₂ production. Under normal aerobic conditions, cells metabolize glucose through the tricarboxylic acid (TCA) cycle in the mitochondria, producing CO₂ as a byproduct. However, in tumor cells, glucose is primarily converted to pyruvate and then reduced to lactic acid, bypassing the TCA cycle and thereby reducing CO₂ generation
Presence of Lactic Acid in the Extracellular Environment	The lactic acid produced by tumor cells is excreted into the extracellular environment, where it dissociates into lactate and hydrogen ions (H⁺). This dissociation contributes to the acidification of the tumor microenvironment, creating weakly acidic conditions. In healthy tissues, the buffering of lactic acid can release CO₂, which then diffuses into red blood cells and enhances the Bohr effect, facilitating the release of oxygen from hemoglobin
Acidic pH and Intracellular Oxygen Levels	In the tumor microenvironment, however, the lack of significant CO₂ production means that this buffering process does not generate CO₂ to enhance the Bohr effect. Consequently, the mildly acidic pH of the tumor microenvironment impairs the release of oxygen from hemoglobin, as there is no sufficient CO₂ to shift the oxygen dissociation curve. This impairs the diffusion gradient necessary for oxygen to efficiently enter tumor cells, leading to intracellular hypoxia. This decreases the release of oxygen from hemoglobin, contributing to intracellular hypoxia by impairing the diffusion gradient necessary for oxygen to efficiently enter tumor cells. This hypoxic condition sustains the reliance on anaerobic glycolysis, perpetuating the cycle of lactic acid production and extracellular acidification

Carbon dioxide (CO_2_) can play a role in regulating the acidic microenvironment of the tumor, though its effectiveness and impact are influenced by several factors, including genetic influences, intracellular pH changes, intracellular oxygen levels, and the activation of remaining mitochondria.

**Production and Presence of Carbon Dioxide in Tumors.** In typical cells, CO_2_ is produced as a byproduct of aerobic respiration in the mitochondria. However, tumor cells primarily rely on anaerobic glycolysis, which significantly reduces the production of CO_2_. This metabolic shift leads to a decrease in mitochondrial CO_2_ production, resulting in an altered role for CO_2_ in the tumor microenvironment compared to normal tissues. When the amount of CO_2_ is amplified in the tumor microenvironment through a protocol exposing the patient to an atmosphere of two%–5% CO_2_, the role of CO_2_ in regulating pH starts to take effect. This process occurs through the formation of carbonic acid (H_2_CO₃), which amplifies the buffering capacity. When CO_2_ in aqueous environments reacts with water, it forms H_2_CO₃, which then dissociates into bicarbonate (HCO₃⁻) and hydrogen ions (H⁺). This reaction is reversible and helps buffer changes in pH by either consuming or releasing H⁺, depending on the prevailing conditions. The presence of CO_2_ and its conversion to H_2_CO₃ provide a buffering system that can modulate the pH of the tumor microenvironment. This buffering action can help neutralize excess acids, such as lactic acid produced by glycolytic tumor cells, thereby mitigating extreme pH changes. Böning et al. (1991) documented that the influence on the Bohr effect caused by the release of lactic acid during physical exercise is entirely dependent on the buffering effect of CO_2_.

**Influence of CO_2_ on Oxygen Levels and Mitochondrial Activation.** The amplification of the Bohr effect in the region by buffering lactic acid, in turn, enhances an increase in oxygen levels and mitochondrial activation, indirectly influencing the presence of intracellular oxygen. Increasing the availability of CO_2_ and its subsequent conversion to bicarbonate can facilitate the transport and utilization of oxygen within tumor cells. Higher intracellular oxygen levels can shift the balance from glycolytic metabolism to more oxidative processes.

The activation of remaining mitochondria in tumor cells can also, in theory, be possible. These mitochondria can be activated in the presence of higher oxygen levels and appropriate genetic regulation. This can enhance oxidative phosphorylation and reduce reliance on glycolysis, potentially decreasing the production of lactic acid and altering the microenvironment. Of course, such activation is directly dependent on the quantity of remaining mitochondria in the tumor cell, which in turn is dependent on the genetic characteristics of each tumor type.

**Genetic Influences and Cellular Responses.** Certain genetic factors in tumor cells can influence the response to changes in intracellular oxygen levels. For example, nuclear genome-derived circular RNAs (circRNAs) like circPUM1 can localize in mitochondria and regulate oxidative phosphorylation. Gong et al. (2022) described that knockdown of circPUM1 results in lower intracellular oxygen concentration, downregulated oxidative phosphorylation, decreased mitochondrial membrane potential, increased ROS generation, and shrinking of mitochondria. This regulation is crucial for maintaining mitochondrial function and can be influenced by intracellular oxygen levels, indirectly influenced by the presence of CO_2_ in the extracellular environment.

**Acid-Base Balance and the Bohr Effect.** The presence of CO_2_ in the extracellular environment affects the acid-base balance through the formation of carbonic acid and bicarbonate, which can influence the pH. The Bohr effect, where CO_2_ and H⁺ reduce the affinity of hemoglobin for oxygen, facilitates oxygen release to tissues, indirectly affecting intracellular oxygen levels. However, this effect is more complex in tumor environments due to altered metabolic pathways.

The increase in intracellular CO_2_ can lead to the formation of H_2_CO₃ and subsequently HCO₃⁻ and H⁺, which can alter the intracellular pH. The tumor cells’ ability to manage these changes is critical for their survival and proliferation. Additionally, the weakly acidic microenvironment outside the cell can affect the intracellular pH by impacting the exchange and buffering systems within the cell.

Thus, it is possible to conclude that while carbon dioxide has the potential to regulate the acidic microenvironment of the tumor through its role in forming carbonic acid and buffering pH changes, its effectiveness is influenced by the metabolic and genetic characteristics of the tumor cells. The inherent metabolic shift in tumors towards glycolysis reduces CO_2_ production, limiting its direct impact. However, genetic factors and the potential to increase intracellular oxygen levels and activate remaining mitochondria suggest that CO_2_ can modulate the microenvironment to some extent. This regulation may help buffer the acidity and potentially alter the tumor cells’ metabolic dependencies, offering a nuanced approach to managing the tumor microenvironment.

## 4 Discussion

The insights gleaned from our results lay the foundation for a deeper discussion. We delve into how these findings correlate with and expand upon existing knowledge, driving forward the conversation on CO_2_'s therapeutic frontier. The findings presented provide a nuanced view of CO_2_’s role within the tumor microenvironment. We now discuss these results in the context of existing literature and their potential to revolutionize oncological therapies, revisiting our initial hypotheses and expanding on the theoretical framework introduced earlier. Reflecting on the results, we delve into the potential of CO_2_ modulation within the tumor microenvironment as a groundbreaking approach to cancer therapy. This discussion builds upon our initial exploration of CO_2_'s roles and their implications for treatment, as introduced, ensuring our conclusions directly stem from the foundational insights presented at the outset. In cancer research, manipulating CO_2_ shows promise in influencing the tumor microenvironment, as indicated by the 18 CSFs likely to be affected directly or indirectly. Modulating the tumor microenvironment’s pH via carbonic acid/carbon dioxide may significantly impact enzymatic activity, including heme oxygenase. This could affect tumor cell proliferation, survival, and migration, as well as the immune response. Adjusting the pH may improve immunosurveillance effectiveness and boost immunotherapy. Additionally, an acidic environment can promote angiogenesis and metastasis, implying that increasing the CO_2_ availability to alter pH may help limit tumor growth and dissemination. The production of CO_2_ in the TME can amplify these effects, modulating pH and promoting an environment that favors cell survival and tumor progression. Therapeutic strategies targeting pH regulation and HO-1 inhibition may offer promising approaches to disrupt these mechanisms and promote antitumor immune responses. Therefore, integrating knowledge about the hormetic potential of CO_2_ with HO-1 activity can provide new perspectives for the development of anticancer therapies.

Indirect evidence supporting the hypothesis that increasing CO_2_ in the tumor microenvironment could be a valid medical approach was identified in scientific literature. Liao et al. (2024) demonstrate that modifying the extracellular pH of the tumor microenvironment has an antitumor effect. They explain that the acidic tumor microenvironment is primarily caused by lactic acid production rather than CO_2_, due to abnormal mitochondrial activity in tumor cells. However, the role of pH regulators, such as monocarboxylate transporters (MCTs), carbonic anhydrase IX (CA IX), and Na+/H+ exchangers (NHE1), is critical in maintaining pH balance. These regulators can be overwhelmed by excessive acid production, which perpetuates an acidic environment. This suggests that manipulating these pH regulators, potentially through CO_2_ modulation, might help control acidification and, consequently, tumor invasion, migration, and drug resistance. The study by Ciccone et al. (2020) highlights that the inhibition of CA-IX induces antitumor effects more directly related to the regulation of biochemical and molecular processes within cancer cells. Recently, Cheng et al. (2024) stated that inhibitors of carbonic anhydrase IX (CA-IX) demonstrate inhibitory cancer activity *in vitro*. These studies provide laboratory data closely aligned with what this research aims to demonstrate, as the inhibition of CA-IX results in an increase of carbon dioxide in the tumor microenvironment as a major result. The findings from both studies indirectly underscore the potential therapeutic implications of manipulating CO2 levels in the tumor microenvironment, thereby supporting the broader hypothesis of harnessing the hormetic potential of CO2 for innovative cancer treatments, providing further impetus for research exploring this approach. De Palma et al. (2017) describe the tumor microenvironment as characterized by hypoxia, low pH, low glucose, and high ROS. They find that pH changes influence tumor angiogenesis and immune responses, linking the acidification of the TME to enhanced tumor progression. This implies that interventions aiming to regulate TME pH, possibly through CO_2_ modulation, could enhance anti-angiogenic and immunological responses, presenting new therapeutic strategies. Persi et al. (2018) highlight the importance of intracellular pH (pHi) in cancer metabolism, proposing that modulating pHi could be a novel therapeutic strategy. The presence of CO_2_ in the tumor microenvironment could influence intracellular oxygen influx, which in turn might contribute to rearranging pHi by stimulating existing mitochondrial activity and reducing lactic acid production. This complements strategies targeting extracellular pH and suggests that combining these approaches with CO_2_ modulation could enhance anti-cancer treatments, particularly for aggressive cancers, by inhibiting tumor cell proliferation and survival. Takano et al. (2023) document that CO_2_-induced extracellular pH decrease activates CREB and upregulates TGF-β1, influencing enzymatic activity involved in extracellular matrix production. This supports the idea that CO_2_ can modulate crucial signaling pathways in the TME. Furthermore, Niland et al. (2021) describe how matrix metalloproteinases (MMPs), particularly MMP-14, play a crucial role in tissue remodeling and cancer progression by degrading extracellular matrix components and influencing cell-matrix communication. Given that pH changes can affect MMP activity, the modulation of CO_2_ levels in the TME might indirectly impact the proteolytic activity of MMPs, providing additional support for the potential therapeutic role of CO_2_ in cancer treatment. With these findings in hand, in addition to modulating the tumor microenvironment’s pH, CO_2_ can potentially affect the activity of key enzymes, impacting various biological processes. Further, CO_2_ may influence activities such as autophagy, and pathways involving sirtuins and AMPK, which are essential for cellular stress response and energy homeostasis. By modulating these pathways, CO_2_ could affect cell survival and alter the tumor microenvironment, consequently impacting tumor growth and progression.

Whether this hypothesis can become a reality will depend on several critical factors, including funding availability, forming a multidisciplinary team with the required expertise, and gaining access to advanced research infrastructure. The inherent difficulties in translating preclinical findings to clinical applications due to disparities between experimental models and the complexity of human biological systems pose additional challenges. Despite the ambitious and promising nature of the medical hypothesis, its success hinges on thoughtful planning, ample resources, and collaboration among scientists, clinicians, and research institutions. Adapting the research plan based on interim results and newfound challenges will also be crucial for success (Chesbrough, 2003; Flessa and Huebner, 2021).

The controlled use of CO_2_, either through inhalation or other forms of administration, may be a viable strategy to modulate this microenvironment, augmenting conventional therapies like radiotherapy, chemotherapy, and immunotherapy. This highlights the importance of CO_2_ balance due to the central role of carbonic acid in regulating pH in body fluids. The enzyme carbonic anhydrase, which facilitates CO_2_’s reversible conversion to carbonic acid, is key in this process. Alterations in CO_2_ levels can affect the acid-base balance and the concentration of ROS (Garcia and Ramirez, 2017; Hoiland et al., 2019; Hall and Hall, 2020).

Having established the groundwork through our systematic analysis, we now turn to the implications of these findings, exposure to a CO_2_-enriched environment (3%–5%) could potentially affect cancer progression by modulating pathways. In September 2023, the AuBento Institute—Center for Teaching, Clinic, and Research in Orthomolecular and Integrative Practice, affiliated with the Federal University of Santa Maria, was inaugurated as a business model managed by the value-based healthcare tool to serve as a case study. Since its inauguration, the clinical practice has provided promising results that may encourage further exploration of this hypothesis. For instance, in three documented cases, integrative approaches, including the hormetic therapeutic potential protocol of carbon dioxide in oncological treatments, have been applied. The first case involves a 64-year-old patient with stage IV prostate cancer post-surgery, who experienced an increase in PSA levels (6.07) at the end of October. Approximately 6 months after the integrative protocol was applied, the PSA level dropped to 0.6, with no systemic signs of disease activity. Another patient, a 72-year-old with mantle cell lymphoma, had an estimated survival of 15 days according to local oncologists. After adhering to the integrative protocol, the patient has survived for more than 8 months, with no systemic signs of disease activity. A 48-year-old woman with a 1.1 cm nodule in the left upper quadrant of her breast, diagnosed with invasive ductal carcinoma in the right breast, started the integrative protocol. Upon review 45 days later, the nodule was no longer detected on bilateral breast MRI, with no systemic signs of disease activity. At no point are patients informed that their cancer is cured; rather, they are told that their biological terrain has adjusted and that an oncostatic effect is being achieved. Patients are advised to continuously maintain the integrative practices. The current aim of this research is to be a review article, supporting the presented hypothesis directly or indirectly with the available scientific literature. Therefore, delving into the details of these treatments that provide empirical data is beyond the scope of the current article. However, these clinical cases offer preliminary empirical support for the proposed hypotheses and suggest that further controlled studies and experimental designs are warranted to validate these findings comprehensively. Persistent research is needed to fully understand the therapeutic potential of CO2 in cancer treatment, challenging conventional paradigms and offering new paths for disease prevention and treatment. Nevertheless, no experimental model could validate this research through *in vitro* studies. This limitation is due to the difficulty of an *in vitro* model, such as a cell culture, to accurately replicate all the complex adaptive processes triggered by the human body in response to breathing a CO2-enriched atmosphere.

Building on the insights from our methodological approach, under WP5, we proposed a protocol used in our integrative approaches and suggested for clinical research that involves evaluating participants’ physiological adaptation to increased CO_2_ levels. Participants, chosen based on specific inclusion criteria, will be exposed to an environment enriched with a gradually increasing concentration of CO_2_ (starting at 1% and possibly reaching up to 5% based on individual tolerance). This exposure will occur for extended periods, ranging from 8 to 24 h daily, over a full week, to stimulate a standard adaptive response rather than an immediate one. Although CO_2_ plays crucial roles in human physiology, its regulation and the potential negative impacts associated with elevated CO_2_ levels must be carefully considered for a balanced understanding. Therefore, it is essential to consider the hormetic potential of carbon dioxide and the importance of gradual exposure to increasing concentrations as outlined in the suggested clinical protocol. Symptoms at exposures above 5% in the atmosphere can include confusion, lethargy, respiratory difficulty, and in severe cases, it can lead to coma and death. However, the initial reaction triggered when CO_2_ levels in the blood rise above normal can lead to respiratory acidosis. This condition occurs when CO_2_ accumulates and reacts with water in the blood to form carbonic acid (H_2_CO₃), which dissociates into bicarbonate (HCO₃⁻) and hydrogen ions (H⁺), resulting in a decrease in blood pH. Nevertheless, with prolonged exposure—understanding the high controlled exposure level of CO_2_ as a stress—the body tends to compensate, potentially bringing about the expected clinical benefits.

Besides all the data presented, the study aims to stimulate the exploration of the potential of stress-induced telomere signaling with hormetic effects, a concept originally highlighted by Jacome Burbano and Gilson (2021). In this case, the stress is predefined high levels of CO_2_. The body responds to continuous exposure to high levels of CO_2_ by attempting to restore homeostasis. This is because a CO_2_-rich environment triggers a stress response in the body that disrupts cellular, systemic, and organismal homeostasis. In the early stages, the increase in CO_2_ exposure and subsequently higher carbonic acid concentration may alter the body’s acid-base balance and possibly increase oxidative stress. Oxidative stress can accelerate telomere shortening and damage DNA. However, the body’s antioxidant systems, buffer systems, and DNA repair mechanisms can consistently restore homeostasis despite ongoing exposure to elevated CO_2_ levels. Building upon this understanding, this adaptation might require significant adjustments to preserve genomic stability. Our theory is that physiological adaptation to increased exposure to CO_2_ and carbonic acid could result in changes in the expression of genes associated with oxidative stress metabolism, DNA repair, and telomere maintenance. For instance, the activity of telomerase, a telomeric DNA-adding enzyme, might be modulated as a response to the altered environment, influencing DNA’s regenerative capacity. Therefore, long-term exposure to a high CO_2_ environment and the related increase in the body’s carbonic acid concentration might impact genomic stability, aging, and disease susceptibility. The body’s capability to adjust and control telomere maintenance and repair in response to these environmental challenges is the essence of CO_2_’s hormetic effects. This, ultimately, would be the scientific validation of statements such as Henderson’s (1940) claim, “CO_2_ is the primary parahormone of the whole body.

As suggested by our results, the proposed clinical protocol offers a novel approach to studying the impacts of long-term exposure to CO_2_-rich environments on human physiology. It specifically examines the hormetic potential of CO_2_ and possible therapeutic implications. However, logistical considerations such as creating a controlled environment for CO_2_ exposure, recruiting willing participants for a demanding regimen, as well as obtaining necessary approvals from a research ethics committee to ensure ethical and regulatory compliance are clear necessities. Our discussion underscores the significant implications of manipulating CO_2_ and carbonic acid for cancer therapy. Drawing upon these insights, we conclude by reflecting on the study’s contributions to the field and outlining avenues for future research. Our discussion elucidates the transformative potential of CO_2_ manipulation in oncology, setting the stage for concluding reflections on this journey from theoretical exploration to tangible therapeutic prospects.

## 5 Conclusion

In conclusion, drawing from the evidence gathered and analyzed, we ascertain that meticulous manipulation of CO_2_ and carbonic acid in the tumor microenvironment holds promising therapeutic potential. This completes our research journey, bridging the introductory premise with empirical findings and theoretical discussions. Building on this foundation, our medical hypothesis proposes an innovative strategy to significantly alter oncological treatment approaches by exploring the impact of CO_2_ and carbonic acid on the tumor microenvironment. Planned pilot clinical studies will further examine hypercapnia’s effects on cellular oxygenation and tumor environment dynamics. This critical initial phase aims to establish a robust understanding that can inform and guide future oncological therapies, potentially unveiling new treatment avenues through enhanced oxygen penetration into tumor cells by increased carbonic acid.

Transitioning this hypothesis into clinical application necessitates numerous careful steps involving thorough preclinical studies and the development of biomarkers to gauge intervention efficacy. It is also crucial to design clinical trials with defined inclusion and exclusion criteria and precise endpoints, bearing in mind the ethical implications of altering cancer metabolism and potential side effects.

Manipulating the tumor microenvironment using carbonic acid offers a novel perspective for improving cancer therapies and necessitates meticulous investigation. This groundbreaking approach in oncology underscores the value of interdisciplinary and collaborative methods, pledging transformative insights and considerable progress in the cancer fight. To conclude, the study of CO_2_ and carbonic acid’s role in the tumor environment presents a potentially groundbreaking approach in oncology, offering new and more effective strategies for tackling cancer. This concept, which carefully transitions from theory to clinical practice, promises to enhance the field of oncology by paving unparalleled paths for cancer treatment development, all while acknowledging current experimental models’ limitations in fully understanding physiological adaptations to continuous CO_2_-enriched atmosphere stressors.
